# A Qualitative Research Study to Explore Perspectives From Individuals Involved in the ROCKET (Reading for Older People, Connecting With Kids and Enjoying Time Together) Intergenerational Reading Project

**DOI:** 10.1192/bjo.2025.10217

**Published:** 2025-06-20

**Authors:** Rachel Rice, Ursula Shepherd, Lindsey Cameron, Lisa Dikomitis, Joanne Rodda

**Affiliations:** 1 KMPT, Kent, United Kingdom; 2 KMMS, Canterbury, United Kingdom; 3 University of Kent, Canterbury, United Kingdom; 4 KMPT, Canterbury, United Kingdom

## Abstract

**Aims:** Intergenerational programmes provide meaningful social contact for older people, and address negative stereotypes of ageing in children. Reading aloud improves language acquisition and literacy in children, however in regions of socio-economic deprivation, many children lack this opportunity. The aim of this qualitative research was to explore the experiences and perspectives of teachers and volunteers involved in an intergenerational reading project within a primary school in an area of high socioeconomic deprivation.

**Methods:** In the ROCKET project, senior volunteers (aged >75) were supported to join an established volunteer reading scheme in a primary school with the support of an existing volunteer. The students who took part in the project were those identified by teachers to have the greatest need for support with reading. This qualitative study incorporated semi-structured interviews with teachers and volunteers in June–August 2024, a year after the project began. Seven participants took part in an interview, including four teachers, an existing volunteer lead and two volunteers over the age of 75 years old. Videos were audio-recorded and transcribed before thematic analysis by two of the authors.

**Results:** Thematic analysis identified six subthemes: improved reading ability in children, children’s social development, reduced loneliness, volunteers’ sense of purpose/enjoyment and future potential of ROCKET and challenges to overcome.

Volunteers valued the social interaction, the sense of purpose, enjoyment and improved confidence that participating provided them. The teachers described students as benefiting from improved confidence and reading ability, and the positive impact of meeting people outside of their usual social sphere. Participants were optimistic about the future potential of ROCKET and felt it could expand beyond literacy to other subjects such as maths or comprehension, and include other year groups. They also felt that communication could be improved between teaching staff and volunteers involved within the project.

**Conclusion:** Data from this small qualitative study suggests that the ROCKET project has the potential to improve student’s literacy skills and confidence, and to positively impact volunteers through reduced loneliness, a sense of purpose and being a part of a community. Participants felt that improved communication between volunteers and teacher would enhance its success, and that there was scope to broaden the project to include other school subjects and a wider range of students.

